# Molecular Research on Platelet Activity in Health and Disease 3.0

**DOI:** 10.3390/ijms23105530

**Published:** 2022-05-16

**Authors:** Maria Valeria Catani, Isabella Savini, Valeria Gasperi

**Affiliations:** Department of Experimental Medicine, Tor Vergata University of Rome, 00133 Rome, Italy; savini@uniroma2.it

## 1. Review Articles

Zhao and Devine [1] exhaustively provide an update on in vitro storage characteristics and in vivo transfusion effects of cold-stored platelets. They provide an overview of the most modern investigative tools, including proteomics and metabolomics, for studying effectiveness, safety, and shelf-life of cold-stored platelets, in order to reevaluate their application in clinics.

Shevchuk and co-workers [2] describe current platelet proteomic technologies, comparing two-dimensional gel electrophoresis with the most recent liquid chromatography (LC) mass spectrometry (MS)-based proteomic approaches, including the newly appeared trapped ion mobility spectrometry (TIMS). What emerges is that platelet proteomics can be useful in addressing basic research questions, as well as in improving platelet transfusion medicine and therapeutic management of platelet dysfunction.

Ostermeier and collaborators [3] review extracellular vesicle (EV)-based therapy for reducing viral infections and inflammation. They critically discuss advantages and limits of this novel therapeutic approach. Current knowledge on platelet immunobiology and effector functions, and current literature on platelet interactions with specific viral diseases (such as human immunodeficiency virus-1 (HIV-1), dengue virus (DENV), influenza A virus (IAV), and severe acute respiratory syndrome coronavirus 2 (SARS-CoV-2)), leads authors to speculate as to how new therapeutic molecules might be encapsulated in platelet EVs to target hard-to-reach biological areas in viral diseases.

## 2. Original Articles

Casagrande and co-workers [4] focus on molecular mechanisms underlying negative impact of platelets on ovarian tumors. They show that in vitro treatment of ovarian cancer cells with activated platelet releasates increases formation of multicellular tumor spheroids and proliferation of ovarian cancer stem cells, thus decreasing cytotoxic and pro-apoptotic effects of different chemotherapy drugs. Detrimental effects of platelet activity are also the object of the study conducted by Dziedzic and colleagues [5], who give further insights on molecular bases responsible for enlarged pro-thrombotic activity of platelets in secondary-progressive multiple sclerosis (MS) patients. They demonstrate that one of the reasons for the elevated risk of ischemic events observed in MS patients may be genetically or phenotypically reinforced mRNA expression of the P2Y12 receptor, in both platelets and megakaryocytes, and enhanced density of these receptors on platelet membrane.

Although a large body of evidence points out to a negative role of platelet activity during pathological conditions, a protective role is also well-established. Accordingly, due to their enriched cargo in bioactive compounds, including growth factors and anti-inflammatory molecules, platelet preparation lysates represent a successful strategy for treatment of inflammatory conditions and regenerative medicine. By investigating the in vitro anti-inflammatory activity of various platelet-rich fibrin preparations (PRFs), Kargarpour and colleagues [6] highlight the species-specificity, as they show that PRFs exert potent anti-inflammatory activity on murine, but not on human, mesenchymal cells. As shown by Sovkova and co-workers [7], preparation protocols also contribute to variability of PRF effects; by comparison of human plasma and/or platelet lysates produced by different methods, they elegantly demonstrate that platelet proteins alone are not sufficient for providing optimal growth and viability of murine fibroblasts and human mesenchymal stem cells. The authors conclude that regenerative medicine application, based on administration of platelet-derived products, should take into account that plasma is necessary for platelet protein activity.

The potential of two phytochemicals, i.e., pterostilbene (a natural stilbenoid occurring in grapes and berries) and rutaecarpine (a bioactive component of the well-known Chinese medicine *Evodia rutaecarpa*), as therapeutic agents against thromboembolic disorders is the focus of two Chinese studies [8,9]. Lin and coworkers [8] find that pterostilbene may be useful in preventing vascular thrombosis: at low concentrations, it strongly inhibits collagen-induced Lyn, Fyn, and Syk phosphorylation, as well as hydroxyl radical formation, and exhibits a strong activity against platelet activation, through the inhibition of integrin αIII/β3-mediated inside-out and outside-in signaling. Huang’s group [9] demonstrates that low doses of rutaecarpine inhibits collagen-dependent aggregation of washed human platelets and reduced microvascular thrombosis in mice; both events depend on inhibition of collagen-mediated stimulation of PLC2/PKC and PIK3/Akt/GSK3β pathways.

Zhou’s group [10] investigates the functional role of megakaryocyte and platelet calcium-sensing receptors (CaSRs) in vascular calcification observed in chronic kidney disease. Both megakaryocytes and platelets express the Ca^2+^ channel ORAI and its activating Ca^2+^ sensor STIM that are associated with platelet activation. These two proteins are upregulated by hyperphosphatemia occurring in chronic kidney disease and the authors reported the beneficial action of MgCl_2_ and the CaSR agonist GdCl_3_ in counteracting this detrimental phenomenon, thus suggesting that pharmacological modulation of CaSR by both Mg^2+^ and Gd^3+^ might restore calcium homeostasis in blood.

Finally, a rapid and reproducible test for routine laboratory identification of platelet activation is described by Watanabe’s group [11], who set-up a DAPI-based protocol for quantifying polyphosphate (polyP), a linear, unbranched polymer of orthophosphate residues, whose release by platelets increases upon activation. The authors show that this time- and cost-saving procedure also reduces bias for reproducible quantification, thus suggesting that polyP levels, easily and economically quantified, could represent a marker of the quality of platelet preparations.
